# The Effect of Cranio-Cervical Artery Stenosis on Glymphatic System Function in Patients with Cerebral Infarction

**DOI:** 10.3390/jcm15062118

**Published:** 2026-03-10

**Authors:** Xin Liu, Huimin Qiao, Cuining Li, Xiangjian Zhang, Yuxiao Gao, Meiling Song, Yatong Wang, Yi Yang

**Affiliations:** 1 Key Laboratory of Clinical Neurology, Ministry of Education, Hebei Medical University, Shijiazhuang 050000, China; 2 Department of Neurology, The Second Hospital of Hebei Medical University, Shijiazhuang 050000, China; 3 Key Neurological Laboratory of Hebei Province, Shijiazhuang 050000, China; 4 Hebei Key Laboratory of Vascular Homeostasis and Hebei Collaborative Innovation Center for Cardiocerebrovascular Disease, The Second Hospital of Hebei Medical University, Shijiazhuang 050000, China; 5 Department of Radiology, The Second Hospital of Hebei Medical University, Shijiazhuang 050000, China

**Keywords:** glymphatic system, cerebral infarction, cranio-cervical artery stenosis, diffusion tensor imaging

## Abstract

**Background/Objectives**: The aim of this study was to investigate the effects of cranio-cervical artery stenosis (CAS) and cerebral infarction (CI) on the function of the glymphatic system (GS). **Methods**: Hospitalised patients with CI and/or CAS were enrolled, along with a control group. A total of 111 participants (62.68 ± 9.85 years; 37% female) were enrolled in this study. GS function was assessed using the diffusion tensor imaging analysis along with the perivascular space (DTI-ALPS) method. The influencing factors and the individual and combined effects of CI and CAS on the DTI-ALPS index were analysed. **Results**: Age (*p* = 0.024), CI (*p* < 0.001), and CAS (*p* = 0.001) were independent predictors of a lower DTI-ALPS index. There were statistically significant differences in the DTI-ALPS index among the four groups (CI, CAS, CI + CAS, control) (F(3, 107) = 91.4, *p* < 0.0001). The DTI-ALPS index was lower in the CI, CAS, and CI + CAS groups compared with the control group (*p* < 0.0001); in the CI group compared with the CAS group (*p* < 0.0001); and in the CI + CAS group compared with the CI group (*p* < 0.05). CI and CAS were found to have a significant interaction effect on the DTI-ALPS index (F(1, 107) = 6.43, *p* = 0.013). **Conclusions**: Aging, CAS, and CI independently impair GS function, with CI having a stronger effect. All three are independent predictors of GS dysfunction. Patients with CAS experience more significant GS dysfunction after suffering CI than patients without CAS. CI and CAS have a synergistic effect on GS impairment.

## 1. Introduction

The glymphatic system (GS) is a system that maintains the stability of the brain tissue environment through the exchange of cerebrospinal fluid and tissue fluid [1]. It uses the perivascular space as a pathway and relies on driving forces such as craniovertebral artery pulsation, respiration, and concentration gradients of blood permeables (e.g., glucose, sodium) [2]. The directed fluid flow mediated by aquaporin-4 (AQP-4) in astrocytic terminal processes enables the performance of functions such as intracellular metabolite clearance, immune responses, and lipid redistribution. Cerebral infarction (CI) instigates a sequence of ischemic injury reactions, including oxidative stress and inflammation, resulting in the accumulation of metabolic toxins and the subsequent occurrence of cytotoxic oedema. The GS is a pivotal conduit for the elimination of metabolic byproducts. However, ischemic injury also disrupts the dynamics and structure of the GS, and the surge in metabolic toxins [3] further exacerbates GS dysfunction.

Cranio-cervical artery stenosis (CAS) has been identified as one of the most common causes of CI, with an incidence rate as high as 46% in stroke or TIA patients. In cases of arterial stenosis, there is an observed decrease in vascular wall compliance, which results in impaired pulsation. It is hypothesised that CI patients with CAS may experience more severe GS dysfunction. Diffusion tensor imaging (DTI) analysis along the perivascular space (DTI-ALPS) is a non-invasive approach to evaluating GS function based on DTIs. This study employs the DTI-ALPS index to clarify the effects of CAS and CI on GS function, thereby providing a theoretical foundation for clinical practice.

## 2. Materials and Methods

### 2.1. Study Population

#### 2.1.1. Study Population Inclusion and Grouping

**Figure 1:**
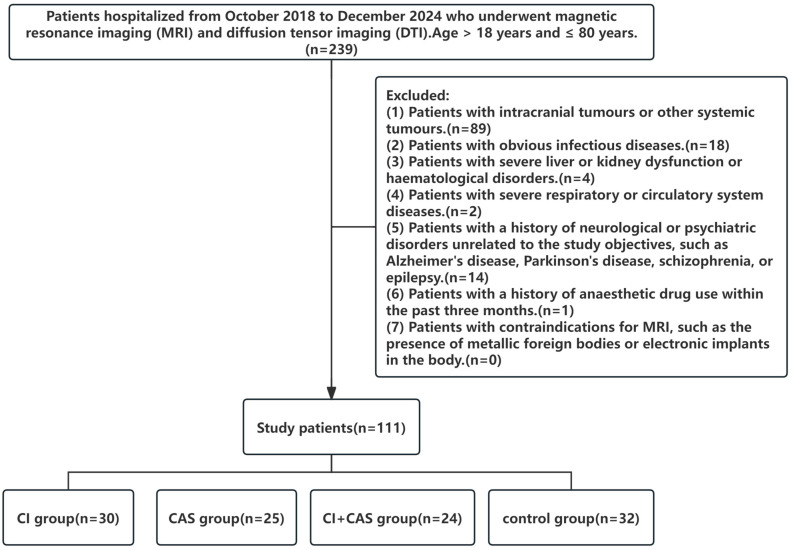
**Flowchart of patient selection:** A total of 239 patients hospitalised from October 2018 to December 2024 who underwent magnetic resonance imaging (MRI) and diffusion tensor imaging (DTI) were included, while 128 patients were excluded. Eventually, 111 patients who met the inclusion criteria were included. CI, cerebral infarction; CAS, cranio-cervical artery stenosis.

This study included patients hospitalised from October 2018 to December 2024 who underwent magnetic resonance imaging (MRI) and DTI. The study subjects were grouped according to the inclusion and exclusion criteria into the following groups: a CI group (cerebral infarction without hemodynamically significant stenosis), a CAS group (cranio-cervical artery stenosis without infarction), a CI + CAS group (combined infarction and cranio-cervical artery stenosis), and a control group. For the details of this process, please refer to Figure 1.

#### 2.1.2. Inclusion Criteria

(1) Age > 18 years and ≤80 years.
(2) CI group: Cerebral infarction without hemodynamically significant stenosis. The diagnosis of CI conforms to the diagnostic criteria in the *Chinese Guidelines for the Diagnosis and Treatment of Acute Ischemic Stroke 2018* and is confirmed by diffusion-weighted imaging (DWI), with no stenosis (≥50%) of the cranio-cervical arteries confirmed by magnetic resonance angiography (MRA) or computed tomography angiography (CTA). Patients must have received an MRI examination within 14 days of CI onset.
(3) CAS group: Cranio-cervical artery stenosis without infarction. Patients with stenosis (≥50%) in one or more of the cranial and cervical arteries calculated using the North American Symptomatic Carotid Endarterectomy Trial (NASCET) method and the Warfarin–Aspirin Symptomatic Intracranial Disease Trial (WASID) method, including the internal carotid artery, middle cerebral artery (M1/M2), and anterior cerebral artery (A1), confirmed by CTA or MRA, without acute CI.
(4) CI + CAS group: Combined infarction and stenosis. Patients with both acute CI and one or more CAS (≥50%).
(5) Control group: Patients without CI or CAS, who do not meet the exclusion criteria and have undergone MRI.

#### 2.1.3. Exclusion Criteria

(1) Patients with intracranial tumours or other systemic tumours.
(2) Patients with obvious infectious diseases.
(3) Patients with severe liver or kidney dysfunction or haematological disorders.
(4) Patients with severe respiratory or circulatory system diseases.
(5) Patients with a history of neurological or psychiatric disorders unrelated to the study objectives, such as Alzheimer’s disease, Parkinson’s disease, schizophrenia, or epilepsy.
(6) Patients with a history of anaesthetic drug use within the past three months.
(7) Patients with contraindications for MRI, such as the presence of metallic foreign bodies or electronic implants in the body.

### 2.2. Data and Methods

#### 2.2.1. Collection of General Data, Blood Markers, and Imaging Markers

General data and blood indicators were collected from the study subjects. Blood parameters were monitored by the hospital’s Laboratory Department, using fully automated blood cell and biochemical analysers. Cranial MRI images and CTA images of the carotid and cervical arteries were collected, including DTI sequences, DWI sequences, fluid-attenuated inversion recovery (FLAIR) sequences, and MRA sequences. The MRI images were acquired using a Siemens MRI machine.

MRI examinations were performed on a 3.0 T MR scanner with a standard head coil. The cranial MRI sequences included T2 FLAIR and DTI sequences. The MRI parameters were as follows: (1) DTI sequence: TA = 6:30, voxel size = 2.0 × 2.0 × 2.0 mm, FOV = 224 mm, slice thickness = 2 mm, slices = 75, TR = 10,800 ms, TE= 90.0 ms, maximum b-value = 1000 s/mm^2^, 30 non-collinear directions. (2) DWI sequence: TR = 6000 ms, TE = 80 ms, voxel size = 1.5 × 1.5 × 5.0 mm, slices = 20, FOV = 240 mm, slice thickness = 5.0 mm with no gap between slices, b-values = 0 and 1000 s/mm^2^, 15 diffusion encoding directions. (3) T2-FLAIR sequence: TR = 9000 ms, TE = 85 ms, voxel size = 0.5 × 0.5 × 6.0 mm, slices = 20, FOV = 240 mm, slice thickness = 6.0 mm with no gap between slices, flip angle = 150 deg. (4) MRA sequence: TR = 25 ms, TE = 3.4 ms, voxel size = 0.5 × 0.5 × 1.0 mm, slices = 120, FOV = 240 mm, slice thickness = 1.0 mm with no gap between slices, flip angle = 20 deg.

#### 2.2.2. Clinical Data and Imaging Post-Processing

The AIP and TyG indices were calculated using the collected clinical data. The AIP index was calculated by using the formula log (Triglycerides (mmol/L)/High-density Lipoprotein (mmol/L)). The TyG index was calculated by using the formula Ln [Triglycerides (mg/dL) × Fasting Blood Glucose (mg/dL)/2].

Magnetic resonance images were processed using DSI Studio software (version Chen May 6 2024). Uniform circular regions of interest were placed on the periventricular plane, and diffusion rate indices in the X, Y, and Z directions were obtained. The DTI-ALPS index was calculated using the following formula:

$$\mathrm{ALPS}\;\mathrm{index}=\frac{\mathrm{mean}(\mathrm{Dxxpr}\;\mathrm{ojn},\;\mathrm{Dxxassoc})}{\mathrm{mean}(\mathrm{Dyypr}\;\mathrm{ojn},\;\mathrm{Dzzassoc})}$$

The diffusivity properties of projection and association fibres along the *x*-axis are denoted by Dxx-proj and Dxx-assoc, respectively. The diffusion behaviour of projection fibres along the *y*-axis is characterised by Dyy-proj, whereas the *z*-axis diffusivity of association fibres is represented by Dzz-assoc. Projection fibres run vertically and are responsible for signal transmission between the cerebral cortex and subcortical structures. Association fibres run horizontally or obliquely and mainly connect different cortical regions within the same cerebral hemisphere.

The DTI-ALPS index was independently measured by a neuroradiologist (with more than 15 years of experience in neuroimaging) who was blinded to the grouping information. To ensure the objectivity of this assessment, (**i**) all imaging data had patient IDs, clinical diagnoses, and grouping labels removed; (**ii**) the assessor only accessed DTI sequence images, avoiding exposure to other sequences that might contain information about infarction or stenosis; (**iii**) the images were sorted by using random numbers; and (**iv**) in DSI Studio software (version Chen May 6 2024), ROIs (regions of interest) were placed according to a unified standard (a 2 × 2 × 4 mm area located in the horizontal plane of the lateral ventricle body, where the fibre crossing area is projected) [4].

#### 2.2.3. Observation Indicators

(1) The correlations of general information, blood indicators, CI, and CAS with the DTI-ALPS index.
(2) Differences in the DTI-ALPS index between the CI, CAS, CI + CAS, and control groups.
(3) Whether CAS and CI have a synergistic effect on the DTI-ALPS index.

### 2.3. Data Processing and Statistical Analysis

Based on previous studies in the literature [5], the sample size estimation was performed using G Power 3.1 software. The parameters were set as follows: ANOVA (fixed effects, omnibus, one-way), effect size f = 0.35 (moderate effect based on prior DTI-ALPS studies), α = 0.05, and β = 0.20 (power = 80%). This calculation indicated a minimum requirement of 100 subjects. Accounting for an estimated 20% dropout rate (e.g., due to image artifacts or exclusion criteria), a total of 120 participants were planned to be enrolled.

The detailed process of effect size estimation refers to Cohen’s formulas. In the classic definition, f = 0.25/0.40/0.60 is defined as a small/medium/large effect size, and 0.35 is considered to be a medium effect.

$$f=\sqrt{\frac{n^{2}}{1-n^{2}}}\;\mathrm{where}\;n^{2}=\frac{SS_{between}}{SS_{total}}$$

Statistical analyses were performed using SPSS 28.0 software (IBM, Armonk, NY, USA) and SPSSPRO. To eliminate multicollinearity, the relationships between the study subjects’ general characteristics and blood indicators and their DTI-ALPS index values were analysed using regularised Ridge regression. Intergroup differences in the DTI-ALPS index among the four groups were analysed using analysis of variance (ANOVA), with pairwise comparisons made between the groups using the LSD method. The effects of arterial stenosis and CI on the DTI-ALPS index were analysed using two-way ANOVA, with further analysis to determine the presence of synergistic effects. A *p*-value of <0.05 was considered to indicate statistical significance.

## 3. Results

### 3.1. Analysis of Factors Influencing the DTI-ALPS Index

The CI, CAS, CI + CAS, and control groups included 30, 25, 24, and 32 cases, respectively, for a total of 111 cases just short of the planned 120 cases. The demographic and clinical characteristics of each group are shown in Supplementary Table S1. The sample size was based on feasibility. Our hospital launched its DTI examination program in 2018, and we included all inpatients who met the criteria during the period from 2018 to 2024. Effect size analysis: The calculated Cohen’s effect size f based on the actual data was 1.60, indicating significant differences among the groups. Under the condition of α = 0.05, the statistical test power was >99.9%, ensuring reliable detection of the group differences. The average time from symptom onset to MRI examination for patients in the CI, CAS, and CI + CAS groups was (4.2 ± 2.7) days.

Ridge regression analysis was used, and, based on the principle of minimising variance expansion factors, λ = 0.182 was determined to analyse the associations among the general data, blood indicators, and DTI-ALPS index values (Table 1). Table 1 shows that age (*p* = 0.024), CI (*p* < 0.001), and CAS (*p* = 0.001) were statistically significant factors associated with the DTI-ALPS index, making them independent predictive factors of a reduced DTI-ALPS index. No other baseline data showed a statistically significant association with the DTI-ALPS index (*p* > 0.05).

### 3.2. Analysis of the Effects of CAS and CI on the DTI-ALPS Index

**Figure 2:**
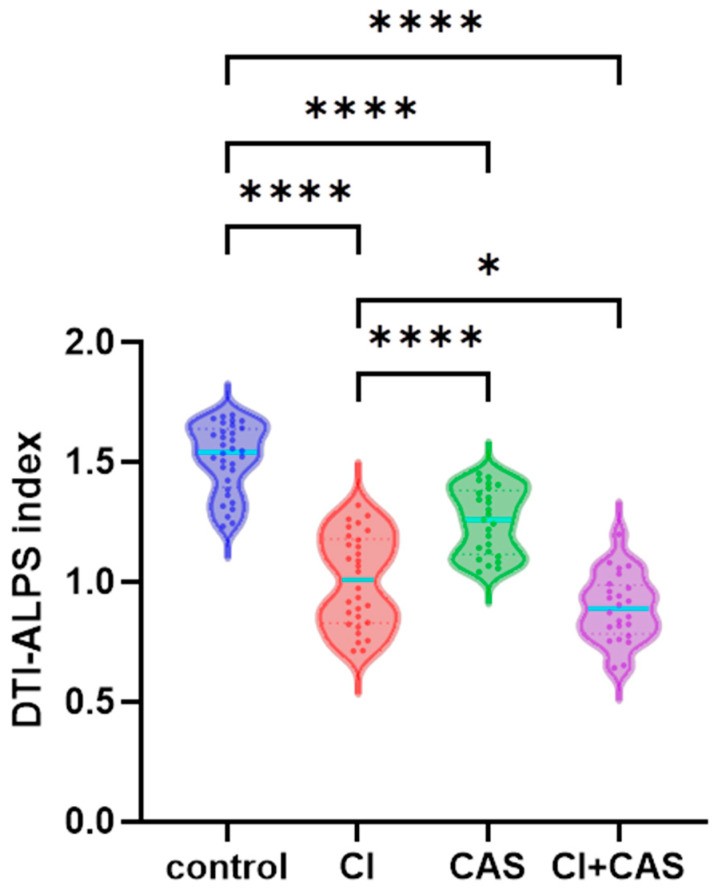
**Comparison of DTI-ALPS index values among the CI, CAS, CI + CAS, and control groups:** The DTI-ALPS index was lower in the CI (*n* = 30), CAS (*n* = 25), and CI + CAS (*n* = 24) groups compared with the control group (*n* = 32) (*p* < 0.001); in the CI group compared with the CAS group (*p* < 0.001); and in the CI + CAS group compared with the CI group (*p* < 0.05). * *p* < 0.05, **** *p* < 0.0001.

A one-way analysis of variance was used to compare the DTI-ALPS index values among the CI, CAS, CI + CAS, and control groups. The results showed that there were statistically significant differences in the DTI-ALPS index among the four groups (F(3, 107) = 91.4, *p* < 0.0001). The DTI-ALPS index was lower in the CI, CAS, and CI + CAS groups compared with the control group (*p* < 0.0001); in the CI group compared with the CAS group (*p* < 0.0001); and in the CI + CAS group compared with the CI group (*p* < 0.05) (Figure 2). Age was found to be an independent predictor of a reduced DTI-ALPS index. To eliminate the influence of age in the statistical analysis, we explicitly regarded age as a covariate in the multivariate analysis. The results confirmed that even after adjusting for age, CI and CAS were still significantly correlated with a reduction in the DTI-ALPS index.

### 3.3. Investigating a Potential Interaction Effect of CAS and CI on the DTI-ALPS Index

**Figure 3:**
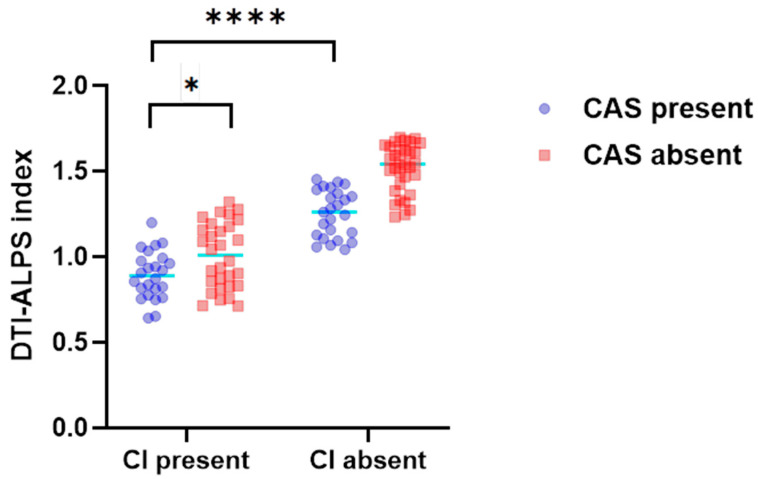
**The influence of CI and CAS on the DTI-ALPS index:** The DTI-ALPS index after CI varied with the presence or absence of CAS. The impact of CI and CAS on the DTI-ALPS index showed a synergistic effect. * *p* < 0.05, **** *p* < 0.0001.

A two-factor analysis of variance showed that CI and CAS had a significant interaction effect on the DTI-ALPS index (F(1, 107) = 6.43, *p* = 0.013). Simple effect analysis indicated that among patients with CAS, those with CI had a lower DTI-ALPS index than did those without CI (*p* < 0.0001), and in patients with CI, those with CAS had a lower DTI-ALPS index than did those without CAS (*p* < 0.05) (Figure 3). This indicated that CI and CAS may have a synergistic effect on damage to GS function.

## 4. Discussion

In this study, the DTI-ALPS index was used as a non-invasive assessment indicator of GS function, and independent predictors of GS damage were identified. It was also found that CAS and CI had a synergistic damaging effect on the GS, with the influence of CI being more significant. Patients with CAS experienced more severe GS dysfunction after suffering from CI. This viewpoint provides a new perspective for clinical intervention in cases of CI, suggesting that early CAS screening and treatment are of great significance.

In recent years, the GS has been revealed as a core mechanism in central nervous system metabolism [6], and it is closely related to the pathological processes of various central nervous system diseases [7], such as stroke, cerebral oedema, and Alzheimer’s disease [8]. Currently, most research results on the GS have come from animal experiments [9]. The prevailing view holds that cerebrospinal fluid enters the brain parenchyma through perivascular spaces (PVSs) around perforating arteries, a process primarily driven by arterial pulsations. Subsequently, cerebrospinal fluid crosses the basement membrane and astrocytic endfeet from the perivascular spaces, enters the brain parenchyma via AQP-4-mediated transport, mixes with interstitial fluid, and is ultimately drained through meningeal lymphatic vessels and arachnoid granulations [10,11]. Accumulating evidence indicates that the elevated extracellular water content observed in cerebral small vessel disease (SVD) is associated with impaired cerebral fluid exchange, PVS dilation, and other typical manifestations of SVD [12]. Previous studies have identified multiple factors influencing the GS, including physiological factors such as circadian rhythms [13] and age [14], as well as pathological factors such as Alzheimer’s disease [15], stroke [16], traumatic brain injury [17], and anaesthetic drugs. This study observed the GS function of patients with cerebral infarction combined or not combined with carotid–cervical artery stenosis. It was found that age, CAS, and CI were independent predictors of GS dysfunction, further supporting and supplementing the previous research conclusions.

In addition, previous GS function monitoring mainly relied on invasive methods, such as injecting contrast agents or tracers into the cerebrospinal fluid through lumbar puncture [18]. These methods have operational risks and are subject to ethical constraints. The DTI-ALPS index, which is obtained through post-processing of the MRI DTI sequence, quantifies the anisotropy of water molecule diffusion in the PVSs, thereby enabling non-invasive monitoring of GS function in this study [19].

### 4.1. The Impact of Age on GS Function

With continued aging, various factors contribute to impaired GS function. Older age has been shown to be associated with an elevated risk of developing arteriosclerosis, reduced vascular wall compliance, and significantly decreased arterial pulsatility [20]. In the aging brain, reactive astrocyte proliferation occurs, and AQP-4 depolarisation takes place. Highly polarised AQP-4 on astrocyte terminal processes is a key component of the GS. The loss of AQP-4 polarisation leads to reduced GS function [21]. Aging also causes inactivation of the Notch3 signalling pathway, alterations in calcium ion concentrations and vascular constriction function, and increased extracellular matrix deposition; this results in cerebral vascular dilation, tortuosity, microaneurysm formation, and reduced cerebral blood flow, thereby impairing GS function [22]. Additionally, elderly patients often have underlying conditions such as systemic inflammation, which may also damage the GS [23]. The conclusions of this study are consistent with those of previous reports.

Importantly, while our univariate comparisons revealed significant differences in the DTI-ALPS index among the groups, age could theoretically confound these associations because it was identified as an independent predictor in the ridge regression model. To address this, we explicitly included age as a covariate in the multivariate analysis. The results confirmed that both CI and CAS remained significantly associated with reduced DTI-ALPS index values even after adjustments for age and other baseline variables. This indicates that the detrimental effects of CI and CAS on glymphatic function are not merely an epiphenomenon of age-related changes. Nevertheless, we acknowledge that residual confounding by age cannot be completely excluded in an observational study, and we have therefore highlighted age as a potential modifying factor in our interpretation of our findings.

### 4.2. The Impact of CAS and CI on GS Function

Animal experiments have shown that cranio-cervical artery pulsation facilitates the introduction of cerebrospinal fluid into the perivascular spaces and brain parenchyma [24,25,26]. This study found that GS function was impaired in patients with CAS, with potential mechanisms including the following: (**i**) CAS often leads to reduced vascular wall compliance and decreased arterial pulsation. (**ii**) CAS increases blood flow resistance, reduces blood flow distal to the stenosis, and weakens the arterial pulsation amplitude. Though some patients may have open collateral circulation, collateral circulation often manifests as low-flow, low-pulsatility slow flow, with a longer blood flow path and significant pressure attenuation, which is unable to fully replace the pulsatility of the main artery. (**iii**) Patients with CAS may experience chronic cerebral hypoperfusion, leading to disrupted polarised distribution of AQP-4 in the astrocyte endfeet and subsequent disruption of GS function [27]. Based on this, we also speculate that patients with CAS are not only prone to CI but also more susceptible to diseases associated with impaired clearance or deposition of metabolic byproducts or abnormal proteins, such as cognitive impairment and cerebral amyloid angiopathy, due to reduced GS function. Recent studies have shown that the ALPS index mediates core memory-related brain structure atrophy during normal aging, which indirectly supports this hypothesis [28]. However, our hypothesis still requires further confirmation through future research.

Previous animal studies have shown that CI transiently impairs GS function [7]. This study confirmed that CI patients have impaired GS function, consistent with the conclusions of animal studies. CI can impair GS function in multiple ways, with the specific mechanisms as follows: (**i**) ischemic damage destroys PVSs, astrocytes, AQP-4, etc., leading to structural damage to the GS; (**ii**) ischemia reduces cerebral blood flow, weakening GS dynamics (arterial pulsation); and (**iii**) ischemic damage produces large amounts of cytotoxic substances, metabolic waste products, inflammatory factors, and other components, exacerbating the burden on GS function and generating a positive feedback effect. Additionally, this study found that CI has a more severe impact on GS function than CAS, suggesting that acute ischemia causes more severe damage to the GS than arterial dynamic abnormalities. Therefore, GS functional repair may serve as a potential target for improving the prognosis of CI. Animal experiments have also confirmed that AQP-4-specific inhibitors can improve GS function [29].

### 4.3. CI and CAS Have a Synergistic Damaging Effect on the GS

Previous studies have found that CI and CAS damage GS function through different pathways. This study, however, indicates that CI and CAS have a synergistic damaging effect on GS function. Specifically, patients with CAS who experience CI exhibit more severe GS dysfunction as compared with those without CAS who suffer from CI. This conclusion may be attributed to the following: (**i**) Patients with CAS experience reduced arterial pulsation, leading to a decline in baseline GS function. Following a CI, the accumulation of metabolic waste and cytotoxic substances further exacerbates GS damage, disrupting the fragile equilibrium of GS function. (**ii**) CAS leads to cerebral hypoperfusion, endothelial cell dysfunction, and low-grade inflammation, making the brain tissue more sensitive to ischemic damage and amplifying GS damage. As such, the synergistic damage to GS function caused by CAS and CI has significant clinical implications, potentially explaining why some patients experience faster deterioration of neurological function and are more prone to complications such as cerebral oedema and post-stroke cognitive impairment.

### 4.4. Innovations and Limitations of This Study

Unlike previous invasive studies, this study conducted a non-invasive assessment of GS function and found that CI and CAS have a synergistic effect in damaging GS function, with the impact of CI being more severe. These findings are significant, suggesting that early CAS screening and treatment may be a new direction for neuroprotection and provide a research direction for exploring the molecular mechanisms of GS.

However, the data for this study were obtained from our hospital, with patients primarily from southern cities in Hebei Province, China; thus, they do not reflect differences across broader regions and populations, resulting in certain limitations of this study. In this study, a single imaging assessor was employed. Although bias was reduced through strict blinding, in the future, multiple centre assessors should be included to analyse measurement variability.

Furthermore, there were significant age differences among the groups, which may confound the interpretation of our results. Although age was included as a covariate in the multivariate analysis, its potential influence cannot be fully excluded.

In addition, the present study did not distinguish between lacunar and non-lacunar ischemic strokes, although these two subtypes differ significantly in their pathophysiology, prognosis, and clinical characteristics [30]. Moreover, further classification of stenosis severity beyond the ≥50% threshold would be helpful to clarify its potential influence on glymphatic function. Future studies should perform more refined data stratification and multicentre validation to better understand the role of the glymphatic system in ischemic stroke.

## 5. Conclusions

Advanced age, CAS, and CI can all cause GS dysfunction, with CI having a more pronounced effect. All three are independent predictors of GS dysfunction. Patients with CAS experience more significant GS dysfunction after suffering a CI than patients without CAS. CI and CAS have a synergistic effect on GS impairment. This synergy exacerbates neurological deficits, hindering recovery and increasing the risk of long-term complications. Early detection and targeted interventions for both conditions are crucial to mitigate GS dysfunction and improve patient outcomes.

## Figures and Tables

**Table 1:** Ridge Regression Analysis of DTI-ALPS Index Predictors.

	Unstandardized Coefficients		Standardized Coefficients	t	*p*
	B	Standard Error	Beta		
Gender	−0.008	0.037	−0.012	−0.207	0.837
Age	−0.004	0.002	−0.124	−2.32	0.024 *
Smoking	−0.011	0.035	−0.019	−0.328	0.744
Drinking	−0.003	0.039	−0.004	−0.071	0.943
Diabetes	−0.019	0.042	−0.024	−0.446	0.657
Hypertension	0.022	0.031	0.037	0.701	0.486
CI	−0.383	0.033	−0.646	−11.782	<0.001 ***
CAS	−0.202	0.031	−0.346	−6.49	<0.001 ***
BMI	−0.003	0.004	−0.039	−0.853	0.397
HCY	0	0.001	0.011	0.209	0.836
UA	0	0	−0.06	−1.105	0.274
Cr	−0.001	0.001	−0.089	−1.634	0.108
HbA1c	0.004	0.014	0.018	0.312	0.756
FBG	−0.001	0.009	−0.007	−0.138	0.891
Na^+^	−0.006	0.006	−0.05	−0.946	0.348
WBC	0.007	0.007	0.059	1.032	0.306
TC	0.005	0.009	0.02	0.601	0.55
TG	0.008	0.014	0.029	0.552	0.583
HDL	−0.04	0.047	−0.046	−0.851	0.398
LDL	0.026	0.016	0.076	1.663	0.102
Lp(a)	0	0.001	0.014	0.264	0.792
HsCRP	−0.005	0.007	−0.042	−0.776	0.441
AIP	0.076	0.085	0.034	0.898	0.373
TyG	0.038	0.041	0.038	0.913	0.365

* *p* < 0.05, *** *p* < 0.001. HCY, homocysteine, UA, uric acid, Cr, creatinine, FBG, Fasting Blood Glucose, HbA1c, glycated hemoglobin, WBC, white blood cell counts, TC, Total cholesterol, TG, Triglycerides, LDL, Low-density lipoprotein, HDL, high-density lipoprotein, Lp(a), Lipoprotein(a), HsCRP, Highly sensitive C-reactive protein, AIP, atherogenic index of plasma, TyG, triglyceride-glucose.

## Data Availability

Data from this study are available from the corresponding author upon request.

## Supplementary Materials

The following supporting information can be downloaded at: https://www.mdpi.com/article/10.3390/jcm15062118/s1, Table S1: Baseline Demographic and Clinical Characteristics of Participants in the Four Study Groups.
