# Supplementary material for: The Effect of Cranio-Cervical Artery Stenosis on Glymphatic System Function in Patients with Cerebral Infarction
Source: J Clin Med. 2026 Mar 10;15(6):2118. doi: 10.3390/jcm15062118 (PMC13026622; doi:10.3390/jcm15062118)
Supplement: Supplementary file 1 [file jcm-15-02118-s001.zip › jcm-4131800-supplementary.pdf]

Supplementary Table S1 Baseline Demographic and Clinical Characteristics of Participants in the Four Study Groups

**Characteristics**	Control group	CI group	CAS group	CI+CAS group	*P* value
Female, n(%)	31.25%	33.33%	48.00%	37.50%	*P*=0.236
Age,years	66.68±5.84	57.50±14.12	61.76±7.67	62.16±10.66	*P*=0.005**
Smoking, n(%)	21.88%	26.67%	36.00%	28.00%	*P*=0.375
Drinking, n(%)	18.75%	16.67%	16.00%	28.00%	*P*=0.378
Diabetes, n(%)	9.38%	16.67%	20.00%	8.00%	*P*=0.175
Hypertension, n(%)	53.13%	30.00%	32.00%	48.00%	*P*=0.381
BMI	23.28±3.17	24.18±4.54	24.38±3.26	24.52±3.76	*P*=0.513
